# HISTORY OF A SUSTAINED AND SUCCESSFUL MODEL OF INTERNATIONAL GERONTOLOGICAL EDUCATION

**DOI:** 10.1093/geroni/igae098.2035

**Published:** 2024-12-31

**Authors:** Steven Zarit

**Affiliations:** The Pennsylvania State University, University Park, Pennsylvania, United States

## Abstract

A successful and sustained study abroad program depends on a foundation built in advance by the faculty of the participating institutions. Three foundational elements contributed to the success of the program, Aging in a Welfare State: What We Can Learn from Sweden, that has been ongoing since 1995. The first and most important foundational element is the relationship between the faculty at participating institutions. In this program faculty of the two initial participating institutions (Jönköping University and Penn State) had worked together for several years prior to developing and offering the program. Another foundational element was long-standing relationships between our Swedish partners and personnel at social and care facilities serving older people in Jönköping. A central goal of the program was that students would learn how care is organized and delivered in Sweden by visiting programs that served older people. Because our Swedish partners had long-established relationships with local care facilities, they were able to arrange visits for students at a range of programs for older persons. Staff at these facilities welcomed us, were open and informative and even invited us to Swedish Fika (coffee and a snack). We were also able to talk with the people using these programs. The visits were the most inspiring part of the class for students because they demonstrated innovative ways of providing care compared to the US. An obvious but important foundational element was having housing, transportation, and other comfort issues in place. These foundational elements were the key to our ongoing success.

